# Tortuosity of branch retinal artery is more associated with the genesis and progress of diabetic retinopathy

**DOI:** 10.3389/fendo.2022.972339

**Published:** 2022-10-06

**Authors:** Yunfeng Song, Zheng Zhou, Henan Liu, Runyu Du, Yaoyao Zhou, Shanshan Zhu, Shuo Chen

**Affiliations:** ^1^ College of Medicine and Biological Information Engineering, Northeastern University, Shenyang, China; ^2^ School of Innovation and Entrepreneurship, Liaoning Institute of Science and Technology, Benxi, China; ^3^ Department of Ophthalmology, Shengjing Hospital, China Medical University, Shenyang, China; ^4^ Department of Endocrinology, Shengjing Hospital, China Medical University, Shenyang, China; ^5^ Research Institute for Medical and Biological Engineering, Ningbo University, Ningbo, China; ^6^ Key Laboratory of Intelligent Computing in Medical Image, Ministry of Education, Shenyang, China

**Keywords:** diabetic retinopathy, vascular tortuosity, branch retinal vessels, fundus imaging, retinal vessel analysis

## Abstract

**Objective:**

The purpose of this study is to investigate the potential of using the tortuosity of branch retinal artery as a more promising indicator for early detection and accurate assessment of diabetic retinopathy (DR).

**Design and method:**

The diagnoses, consisting of whether DR or not as well as DR severity, were given by ophthalmologists upon the assessment of those fundus images from 495 diabetic patients. Meanwhile, benefiting from those good contrast and high optical resolution fundus images taken by confocal scanning laser ophthalmoscope, the branch arteries, branch veins, main arteries and main veins in retina can be segmented independently, and the tortuosity values of them were further extracted to investigate their potential correlations with DR genesis and progress based on one-way ANOVA test.

**Results:**

For both two comparisons, i.e., between non-DR group and DR group as well as among groups with different DR severity levels, larger tortuosity increments were always observed in retinal arteries and the increments in branch retinal vessels were even larger. Furthermore, it was newly found that branch arterial tortuosity was significantly associated with both DR genesis (p=0.030) and DR progress (p<0.001).

**Conclusion:**

Based on this cohort study of 495 diabetic patients without DR and with different DR severity, the branch arterial tortuosity has been found to be more closely associated with DR genesis as well as DR progress. Therefore, the branch arterial tortuosity is expected to be a more direct and specific indicator for early detection of DR as well as accurate assessment of DR severity, which can further guide timely and rational management of DR to prevent from visual impairment or even blindness resulting from DR.

## Introduction

Diabetic retinopathy (DR) is a common microvascular complication of diabetes (1, 2). The number of DR patients worldwide is approximately 95 million (3), and is estimated to reach 156 million by 2045 (4). The local hypoxia caused by hyperglycemia can induce elevated vascular permeability, intraretinal vascular leakage and neovascularization, eventually leading to vision impairment or even blindness (5). However, it is reported that 90% of such vision impairment or blindness can be prevented by the early diagnosis and rational intervention (6, 7). Currently, fundus imaging has been suggested as a regular examination for DR screening at least once a year (8). However, at its early stage, the DR patients rarely suffer from self-perceived vision impairment and the clinical symptoms are often unnoticeable on fundus images, thus its early and accurate diagnosis is still challenging and relies heavily on clinicians’ experience.

Because retinal microvascular damage induced by high-glucose level is typically recognized as the major cause of DR, the retinal microvascular parameters, such as caliber, fractal dimension and tortuosity (9, 10), have demonstrated to be important indicators to quantitatively associate with the early diagnose of DR (11, 12). Among those retinal microvascular parameters, the changes of retinal microvascular tortuosity has been reported to occur earlier than others (13), and is an important hallmark of retinal ischemia that is closely associated with the genesis and progress of DR (14). Numerous studies have been implemented to investigate the correlation between the retinal vascular tortuosity and DR in diabetic patients. Mustafa *et al.* obtained the global tortuosity from 4 DR patients and 16 diabetic patients without DR in DRIVE (15) and STARE databases (16, 17), and higher global tortuosity was observed in DR patients. Moreover, Lee et al. found that increased global tortuosity was also associated with higher level of DR severity, which was investigated on 121 diabetic patients with different levels of DR severity (18). Besides, Sasongko et al. calculated the tortuosity values of retinal arteries and veins separately based on the color fundus photographs of 144 diabetic patients with different levels of DR severity and 80 diabetic patients without DR; only arterial tortuosity showed statistically significant increase between DR group versus non-DR group as well as between mild DR group versus moderate DR group (19, 20). In the above previous studies, the investigations were mainly focused on the tortuosity of main retinal vessels in the absence of branch retinal vessels, due to the relative low contrast of color fundus images and the lack of proper method for branch retinal vessel extraction. However, the abnormalities of branch retinal vessels, causing extracellular matrix protein synthesis and capillary basement membrane thickening, have been proved to be the major pathological basis underlying abnormal main blood vessels in diabetic patients (21, 22). In addition, anomalous angiogenesis can be induced by the nutrient matrix leaked from abnormal retinal vessels, which leads to the acceleration of DR progress (23). Therefore, we infer that branch retinal vessels might have higher correlation with both DR genesis and progress compared to main retinal vessels, in which the tortuosity only from the branch retinal vessels might serve as a more conducive indicator for early diagnosis and accurate assessment of DR. However, to the best of our knowledge, there has been no effort in the literature related to investigating correlation between tortuosity of branch retinal vessels and DR.

In this study, fundus images with good contrast and high optical resolution (∼15μm) were taken by confocal scanning laser ophthalmoscope from 495 diabetic patients without DR and with different DR severity levels. Benefiting from those good contrast and high optical resolution fundus images, the branch arteries, branch veins, main arteries and main veins in retina can be segmented separately, and the tortuosity values of them were further extracted to investigate their potential correlations with DR genesis and progress based on statistical analysis, i.e. one-way ANOVA test. According to the results, the tortuosity of branch arteries showed much larger increment from patients without DR to patients with DR as well as from patients with low level DR severity to patients with high level DR severity. Interestingly, it is newly found that branch arteriolar tortuosity was statistically correlated with both DR genesis (p=0.030) and progress (p<0.001). Therefore, it is expected that the tortuosity of branch retinal arteries can serve as a more promising indicator for the early detection and accurate assessment of DR, which can be helpful for guiding rational management of diabetic retinopathy.

## Materials and methods

### Study participants and fundus image collection

**Figure 1:**
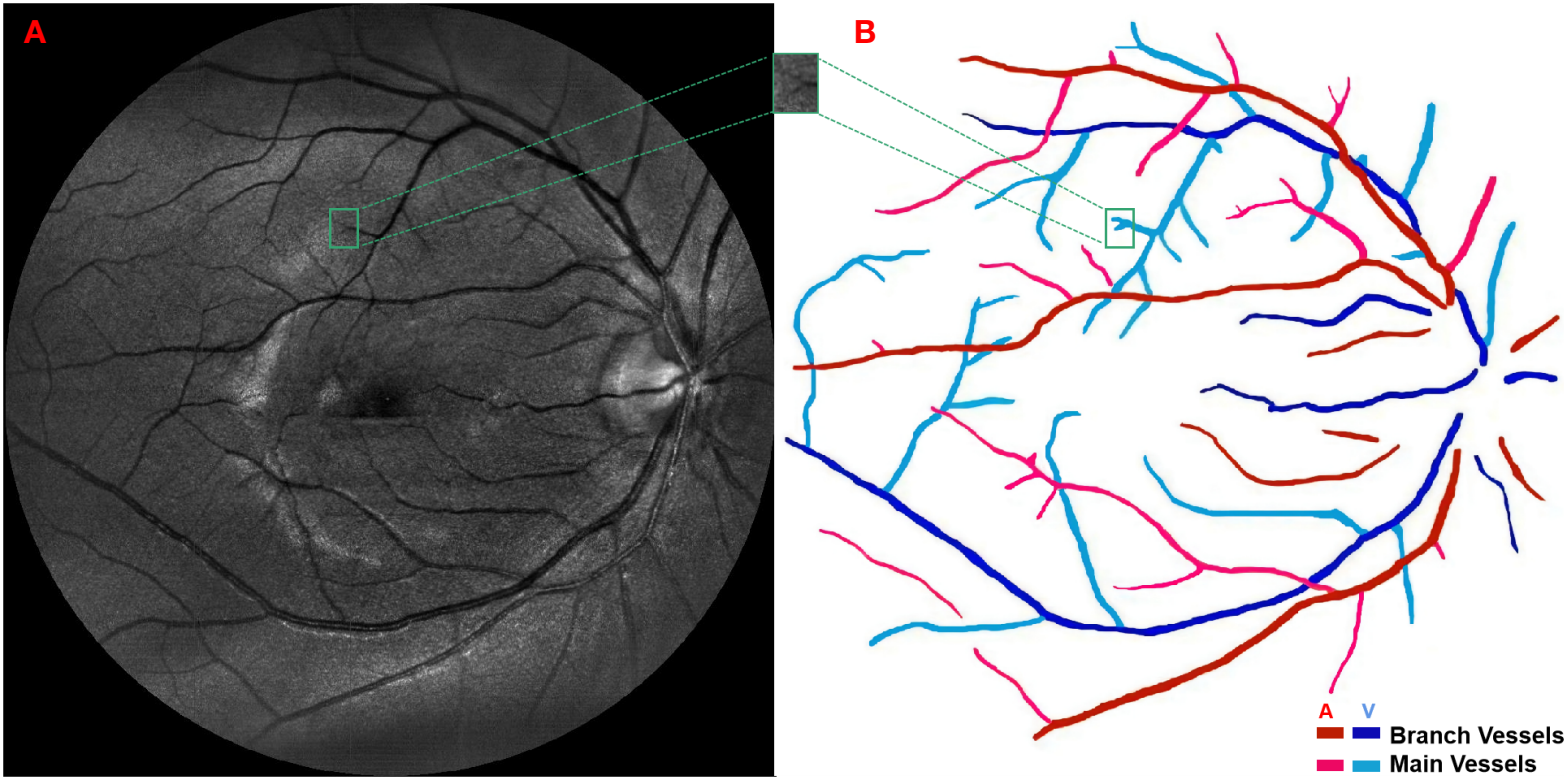
**(A)** A green channel fundus image taken by confocal scanning laser ophthalmoscope and **(B)** the extraction of main arteries, main veins, branch arteries and branch veins.

In this study, a total of 495 patients with type 2 diabetes were recruited from the Endocrinology Department of Shengjing Hospital in China. A pair of fundus images from each patients, i.e., green channel fundus image with 520nm laser and infrared channel fundus image with 780nm laser, were taken by a non-mydriatic confocal scanning laser ophthalmoscope (EasyScan, i-Optics Inc., Netherlands) with 45° field of view. With the employment of confocal optical setup, stray light from the lens and viscous body can be prevented, allowing high optical resolution (~15μm) and good contrast. The green channel fundus image was chosen for further diagnosis and analysis, because of its optimal blood vessel contrast, as show in **Figure 1A**. The fundus image of right eye was preferably chosen for further image processing and analysis; if the fundus image of right eye suffered from low quality (e.g. containing incomplete, blurred regions and/or more than two ungradable vessels), the fundus image of left eye would be chosen instead. The diagnoses, consisting of whether DR or not as well as the DR severity, were given by the ophthalmologists upon the assessment of green channel fundus image following an international clinical DR severity scales (24). More specifically, all patients can be divided into DR and non-DR groups according to whether at least one microaneurysm or hemorrhage can be observed or not; and patients in DR group can be further subdivided into groups with different levels of DR severity, i.e., V1 group (mild non-proliferative DR), V2 group (moderate non-proliferative DR) and V3 group (severe non-proliferative DR and proliferative DR). The patients’ detailed characteristics were listed in **Table 1**. This study has been approved by the medical ethics committee of Shengjing Hospital of China Medical University, and the signed consent forms were obtained from all participants.

**Table 1:** The detailed characteristics of patients in different groups.

Characteristics	Non-DR group (n = 122)	DR group		
		V1 (n = 200)	V2 (n = 139)	V3 (n = 34)
Sex (male/female)	69/53	120/80	80/59	19/15
Age (years)	49.1 ± 15.20	48.5 ± 11.40	56.4 ± 11.50	55.5 ± 10.60
HbA1c (%)	8.9 ± 2.40	8.9 ± 2.30	8.8 ± 2.10	8.61 ± 2.14
Fasting blood sugar (mmol/L)	9.3 ± 3.50	9.3 ± 3.30	9.3 ± 3.70	9.4 ± 4.26
Body mass index (kg/m²)	26.00 ± 4.00	26.0 ± 3.90	25.1 ± 3.10	25.0 ± 2.80

Data are mean ± SD unless otherwise indicated.

### Vascular segmentation, tortuosity extraction and statistical analysis

The green channel fundus images obtained by confocal scanning laser ophthalmoscope were used as the input of Retinal Health Information and Notification System (RHINO) software (25, 26) to extract and differentiate arterial and venous retinal vessels. More specifically, a local normalization was first performed on the original retinal images to alleviate varying contrast problem caused by non-uniform illumination; then, the rotating multiscale second-order Gaussian derivative filters (27) were employed to enhance the retinal vessels and further segment them with proper thresholds; finally, the features of the segmented retinal vessels were extracted, including the mean, standard deviation, median, minimum and maximum of the intensities inside circular regions and the intensity values along the center line of each retinal vessels, and inputted into a logistic regression classifier to differentiate retinal arteries and veins. Those extracted retinal vessels were further segmented into main and branch retinal vessels by a semi-automatic method based on region growing according to the following criterion: only the largest vessels originated from the retinal vessels in optic disk were defined as main retinal vessels while the others were defined as branch retinal vessels, as demonstrated in **Figure 1B**. The outcomes of vessel segmentation and classification were assessed by ophthalmologists and corrected when necessary. Furthermore, the overall tortuosity values of the above four retinal vessel types, i.e., the main artery, main vein, branch artery and branch vein, were calculated based on a published method developed by Bekkers et al. (28) More specifically, the planar retinal vessels were lifted to 3D functions with an additional orientation dimension by convoluting with a specially designed anisotropic cake-wavelet, curvature and confidence values of each location were then extracted by locally fitting exponential curves, and the overall tortuosity of each type of retinal vessels can be derived by the weighted average of the curvatures with confidences as weights (29–31).

The overall tortuosity values of four different retinal vessel types from each patient were served as the independent inputs of the statistical analysis to investigate their potential correlations with DR genesis and progress, respectively. The one-way ANOVA test was implemented to analyze the overall tortuosity values between DR group and non-DR group and the overall tortuosity values among groups with different levels of DR severity, i.e., V1, V2 and V3 groups, and p < 0.05 was considered as statistically significant. The above statistical analyses were conducted by SPSS version 26.0.

## Results

**Figure 2:**
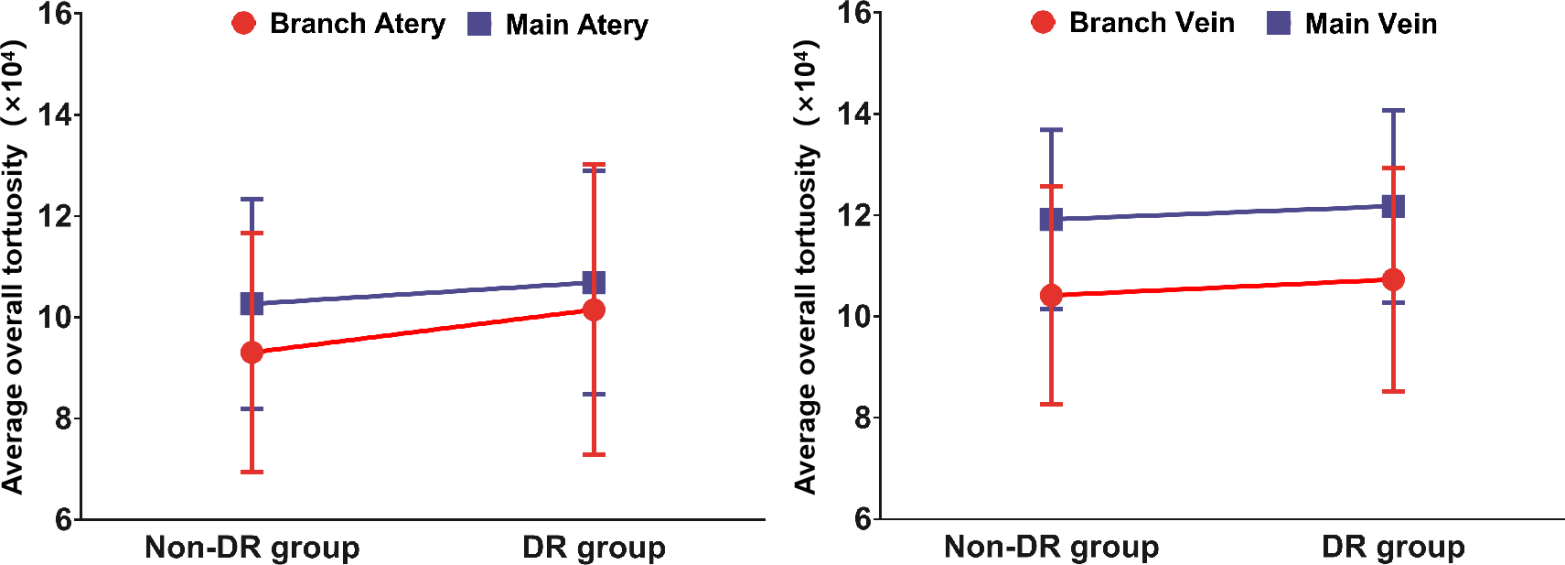
The relationship between retinal vascular tortuosity and DR genesis.

The average overall tortuosity values of main arteries, main veins, branch arteries and branch veins between DR group and non-DR group are listed and plotted in **Table 2** and **Figure 2**, respectively. According to **Table 2** and **Figure 2**, it can be found that the average overall tortuosity of DR group are always larger than those of non-DR group in all types of retinal vessels, which is consistent with the findings of larger global tortuosity in DR patients in other studies (17, 19); furthermore, larger increments of average overall tortuosity are always observed in retinal arteries, and the increments in branch retinal vessels are even larger than those in main retinal vessels; moreover, the statistically significant difference is only observed in branch retinal arteries (p=0.030).

**Table 2:** Comparison of the average overall tortuosity values between DR group and non-DR group.

	Average overall tortuosity (×10^4^)			F value	p value
	Non-DR group	DR group	Difference		
Main artery	10.27 ± 4.16	10.69 ± 4.41	0.42	0.027	0.868
Main vein	11.92 ± 3.53	12.18 ± 3.79	0.26	0.003	0.985
Branch artery	9.31 ± 4.72	10.15 ± 5.73	0.84	4.746	0.030^*^
Branch vein	10.42 ± 4.31	10.73 ± 4.41	0.31	3.210	0.074

*refers to with statistically significant difference.

**Figure 3:**
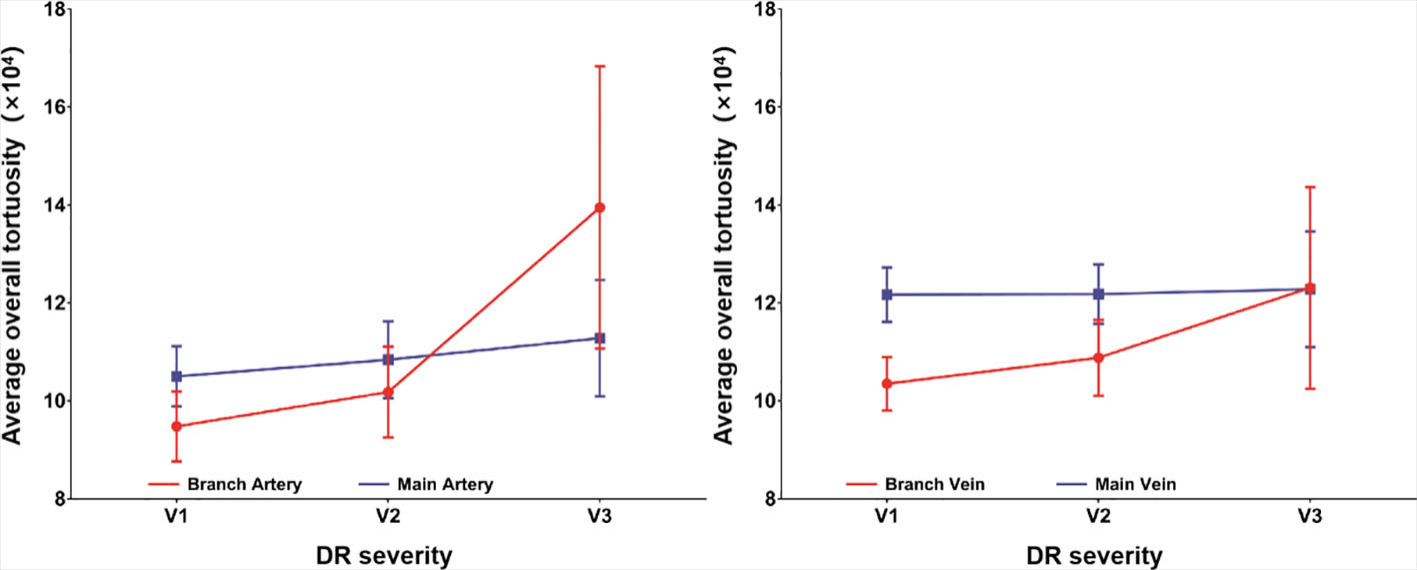
The relationship between retinal vascular tortuosity and DR progress.

The average overall tortuosity values of main arteries, main veins, branch arteries and branch veins among groups with different levels of DR severity (i.e., V1, V2 and V3) are listed and plotted in **Table 3** and **Figure 3**, respectively. According to **Table 3** and **Figure 3**, it can be found that the average overall tortuosity values of all types of retinal vessels increase with higher level of DR severity, which is consistent with the findings of larger global tortuosity in patients with higher level of DR severity in other studies (19, 26), and the increments from V2 to V3 are even larger than those from V1 to V2; furthermore, larger increments of average overall tortuosity are always observed in retinal arteries, and the increments in branch retinal vessels are even larger than those in main retinal vessels; moreover, statistically significant differences are observed in both branch retinal arteries (p<0.001) and branch retinal veins (p=0.049), however, the branch retinal arteries yield much larger statistically significant difference.

**Table 3:** Comparison of the average overall tortuosity values among groups with different levels of DR severity.

	Average overall tortuosity (×10^4^)				F value	p value
	V1 group	V2 group	V3 group	Difference (V3 - V1)		
Main artery	10.50 ± 4.38	10.84 ± 4.66	11.28 ± 3.41	0.78	0.573	0.565
Main vein	12.17 ± 3.98	12.18 ± 3.63	12.28 ± 3.38	0.11	0.011	0.989
Branch artery	9.48 ± 5.11	10.18 ± 5.52	13.96 ± 8.26	4.48	9.271	<0.001^*^
Branch vein	10.35 ± 3.90	10.88 ± 4.61	12.31 ± 5.90	1.96	3.041	0.049^*^

*refers to with statistically significant difference.

## Discussions

Although several previous studies have already investigated the association between retinal vascular tortuosity and diabetic retinopathy, they mainly focused on the global tortuosity of main retinal vessels in the absence of branch retinal vessels, due to the relative low contrast of color fundus images and the lack of proper method for branch retinal vessel extraction. To the best of our knowledge, this study is for the first time that the correlation between the tortuosity of branch retinal vessels and diabetic retinopathy is comprehensively investigated, benefiting from the high contrast fundus images acquired by confocal scanning laser ophthalmoscope and proper branch retinal vessel extraction method. According to the results from 495 diabetic patients, it has been newly found that the tortuosity of branch retinal artery should be more associated with the genesis and progress of diabetic retinopathy, thus the tortuosity of branch retinal artery is expected to serve as a more promising indicator for the early detection and accurate assessment of diabetic retinopathy. More specifically, for both two comparative experiments, i.e., between non-DR group and DR group as well as among different levels of DR severity, larger tortuosity increments were always observed in branch retinal vessels, compared to main retinal vessels; furthermore, statistically significant differences were observed in the tortuosity values of branch retinal arteries for both those two comparative experiments. Thus, the branch arterial tortuosity should be more closely associated with DR genesis as well as DR progress. The potential underlying mechanisms are interpreted as follows.

### DR group versus non-DR group

The larger increment of average overall tortuosity in retinal arteries can be attributed to the fact that blood pressure in arteries is typically higher than that in veins, thus the arterial vascular walls are expected to suffer greater damage due to greater hemodynamic insult. Another reason might be that the arterial occlusion is one of the key pathogenic factors for venous occlusion (32) and commonly occurs earlier, thus such earlier vascular occlusion in retinal artery caused by hyperglycemia is supposed to induce more abnormality in retinal arterial tortuosity. The larger increments of average overall tortuosity in branch retinal vessels can be attributed to the fact that branch vessels with small sizes are more sensitive to hyperglycemia and the subsequent hypoxia-ischemia (33). Based on the joint action of the above two aspects, the largest increment of the average overall tortuosity from non-DR group to DR group is supposed to be found in branch retinal arteries, which further lead to the statistically significant difference between the average overall tortuosity values of those two groups. Therefore, the branch arterial tortuosity is expected to be a more direct and specific indicator for identifying DR.

### Groups with different DR severity levels

The larger increments in arterial and branch vessels can be attributed to the similar facts as discussed above, i.e., higher blood pressure in arteries and vulnerability of branch vessels. The sharper increments of average overall tortuosity from V2 to V3 in branch retinal vessels might be due to the additional vigorous neovascularization (34), which is an unique feature in V3 diabetic retinopathy (35). The underlying mechanism is as follows: hyperglycemia first induces hypoxia-ischemia and further leads to the over-expression of vascular endothelial growth factor (VEGF) (36, 37), i.e., a hypoxia-inducible cytokine to promote neovascularization (38–40), neo-vessels then branch off from pre-existing branch vessels, bend slowly and even cluster in balls (tufts), leading to elevated tortuosity of branch vessels (41), thus the increased tortuosity of branch retinal vessels may signal pathogenic neovascularization induced by hyperglycemia. Recently, Lee et al. have experimentally proved the close relationship between the global tortuosity of retinal vessels and neovascularization in a rat model, in which larger global tortuosity of retinal vessels was observed in rats with more vigorous retinal neovascularization (33). Interestingly, the average overall tortuosity values of branch retinal vessels are close or even much higher than those of main retinal vessels in V3 group, which can be an indirect evidence for the occurrence of hyperglycemia based retinal vascular distortion even during neovascularization. Therefore, the branch vascular tortuosity, especially the branch arterial tortuosity, is expected to be a more direct and specific indicator for assessing DR severity.

## Conclusion

In this study, good contrast and high optical resolution retinal photographs taken by confocal scanning laser ophthalmoscope were used to obtain the overall tortuosity values of main arteries, main veins, branch arteries and branch veins, respectively. Based on the statistical analysis of overall tortuosity, the branch arterial tortuosity should be more closely associated with DR genesis as well as DR progress, and is also expected to be a more direct and specific indicator for early detection of DR as well as accurate assessment of DR severity, which can further guide timely and rational management of DR to prevent from visual impairment or even blindness resulting from DR.

## Data availability statement

The raw data supporting the conclusions of this article will be made available by the authors, without undue reservation.

## Ethics statement

The studies involving human participants were reviewed and approved by The medical ethics committee of Shengjing Hospital of China Medical University. The patients/participants provided their written informed consent to participate in this study.

## Author contributions

YS, SC and SZ contributed to conception and design of the study. HL and RD collected the data. YS, ZZ and YZ performed the data processing and analysis. YS wrote the first draft of the manuscript. All authors contributed to manuscript revision, read, and approved the submitted version

## Funding

This study was supported by National Natural Science Foundation of China (61605025), Fundamental Research Funds for the Central Universities (N2119002) and National Training Program of Innovation and Entrepreneurship for Undergraduates (D202110161215451139).

## Conflict of interest

The authors declare that the research was conducted in the absence of any commercial or financial relationships that could be construed as a potential conflict of interest.

## Publisher’s note

All claims expressed in this article are solely those of the authors and do not necessarily represent those of their affiliated organizations, or those of the publisher, the editors and the reviewers. Any product that may be evaluated in this article, or claim that may be made by its manufacturer, is not guaranteed or endorsed by the publisher.
